# Effect of alendronate on the femoral metaphyseal defect under carbamazepine in ovariectomized rats

**DOI:** 10.1186/s13018-020-02151-1

**Published:** 2021-01-06

**Authors:** Ruotian Zhang, Min Yang, Yang Li, Hedong Liu, Maoxian Ren, Zhou-Shan Tao

**Affiliations:** Department of Trauma Orthopedics, The First Affiliated Hospital of Wannan Medical College,Yijishan Hospital, No. 2, Zhe shan Xi Road, Anhui 241001 Wuhu, People’s Republic of China

**Keywords:** Osteoporotic bone defect, Alendronate, Carbamazepine, Regeneration

## Abstract

**Background:**

The use of antiepileptic drugs and estrogen deficiency put forward higher requirements for bone defect regeneration. The present study investigated the effects of alendronate (ALN) on femoral bone defect in ovariectomized (OVX) rats under the influence of carbamazepine (CBZ).

**Methods:**

One hundred female SD rats at 3 months of age were either sham-operated or OVX and divided into four groups: sham control (CON); OVX control (OVX); ovariectomized rats treated with CBZ via gavage (75 mg/kg/day; CBZ); ovariectomized rats treated with CBZ plus ALN (2 mg/kg/day; CBZ-ALN). A critical-sized femoral metaphyseal bone defect was established in all female SD rats. Animals from the CBZ and CBZ-ALN groups received drugs by gavage the day after bone defect surgery was performed. After the rats were sacrificed, the defected area located in the distal femur was harvested for evaluation by microcomputed tomography (micro-CT), hematoxylin and eosin (HE) staining, and Masson’s trichrome staining. The samples were also analyzed by biomechanics and immunohistochemical evaluation (IHC). Besides, biochemical analysis evaluates all serum samples.

**Results:**

The present study showed that ovariectomy changed the microstructural parameters of bone. The use of CBZ further decreased femur bone mass while treatment with ALN prevented bone loss. Compared to OVX and CBZ groups, CBZ-ALN group promoted bone neoformation and enhanced the ultimate load of the femur bone. However, the group of CBZ-ALN did not return to normal levels compared with the CON group. Besides, we noticed that CBZ-ALN group reduced tartrate-resistant acid phosphatase-5b (Tracp-5b) expression and had no significant effect on the expression of osteocalcin (OCN) and type I collagen (Col-I) in IHC compared with CBZ group. Biochemical analysis results presented that systemic delivery of CBZ showed pernicious effects on bone formation and resorption in ovariectomized rats, with the worse effects on C-terminal crosslinked telopeptide of type I collagen (CTX-1). Besides, a significant decrease in CTX-1 levels was observed in CBZ-ALN group as compared to the group of CBZ.

**Conclusion:**

These results demonstrated that ALN can effectively reverse the effects of CBZ on the microarchitectural properties of bone, and thus can have a positive effect on local bone neoformation in rats with osteoporosis.

**Clinical relevance:**

The dose of 2 mg/kg ALN improves the negative effect of prescription of CBZ at 75 mg/kg and promotes bone neoformation of femoral bony deficits.

## Introduction

Osteoporosis (OP) has become a major clinical problem with increasing human life expectancy. Characterized by reduced bone mass, microstructure destruction, and increased fragility of bone tissue, osteoporosis leads to an increased risk of bone fracture [1, 2]. Bone defect, which is secondary to some diseases such as osteoporosis and malformed bone tissue, requires the formation of new bone to regain healthy morphology and restored function. Although bone tissue has a prominent capacity for self-renewal, if the deficiency is too large, the bone tissue may fail to be thoroughly repaired [3]. Nowadays, with the arrival of the age of population aging, osteoporosis in the elderly poses a major challenge to bone regeneration after fracture or bone defect [4]. Although most studies on osteoporosis have focused on the use of multiple pharmacological agents to prevent fractures, the effects of osteoporosis on fracture and bone defect regeneration have not been well studied up to now [5–7].

Numerous studies have shown that estrogen is an important hormone which has a pivotal role in human [8, 9]. One of the primary causes of postmenopausal osteoporosis is estrogen deficiency, which is closely associated with increased osteoclast activity and bone remodeling. Bone resorption predominance over bone formation results in reduced bone mass and increased bone fragility [10]. In addition, estrogen deficiency caused by ovariectomy leads to delayed bone neoformation possibly due to the expression ratio of the estrogen receptor [11]. Epilepsy is one of the most common serious brain diseases, and it affects more than 70 million people worldwide [12, 13]. Long-term global studies have consistently confirmed that epilepsy patients have a much higher risk of fractures due to a variety of factors, including the risk of falls and the adverse effects of increased frequency, reduced bone strength, or AEDs [14]. People with epilepsy are at a significantly increased risk of osteoporosis and fractures. The obvious focus for the increased fracture risk and the common denominator for most patients with epilepsy is the use of AEDs. In patients associated with epilepsy and postmenopausal osteoporosis, impaired power of bone neoformation may have significant adverse effects on morbidity and quality of life, while also placing a greater financial burden on the patient [15]. Today, the clear focus and common denominator of increased fracture risk in most epileptic patients is the use of AED. When the combination of epilepsy and osteoporosis occurs, the bone mass of patients is already severely low, and the use of antiepileptic drugs will only reduce bone mass further [6]. In order to counteract the adverse effects of AEDs, vitamin D and calcium supplements are often recommended for long-term AEDs users. However, due to the limited clinical data, whether or not to add bisphosphonates still bothers clinicians [16].

Bone is a dynamic tissue endlessly reshaped by the synergistic action of osteoclasts and osteoblasts [17]. Disruption of the balance between the two would have serious consequences [18]. Recent evidence shows that antiepileptic drugs (AEDs) can significantly increase the risk of bone conversion and fracture [19, 20]. Carbamazepine (CBZ) is an iminodibenzyl derivative belonging to the family of traditional enzyme-induced antiepileptic drug that functions in the production of hepatocyte cytochrome P450. It is widely used to treat partial and secondary systemic seizures and has been reported to cause nausea, vomiting, and weight gain as the common side effects, in addition to osteoporotic bone loss [7]. A recent report of AEDS in human bone cells reported that AEDS including CBZ directly regulate the occurrence of human osteoblasts and osteoclasts, which partly explaining some of the potentially harmful effects of AEDS on bone metabolism [21]. Likewise, Parveen et al. [22] pointed to the fact that modulation of wnt inhibitors might be partly involved in contributing to the adverse effects of CBZ on bone. Alendronate (ALN) is a nitrogenous bisphosphonate that is concentrated in active bone remodeling sites in the body. Induction of osteoclast apoptosis is one of the main mechanisms of bisphosphonates [23]. Functionally, ALN can inhibit bone resorption by influencing the formation of osteoclast wrinkle boundaries and regulating the molecular pathways of cytoskeleton [24]. Previous studies have shown that ALN can prevent bone resorption, and ALN therapy increases bone strength by significantly affecting bone mass and bone quality. In addition, ALN may delay the process of cortical bone transformation and have a positive effect on bone density in metaphyseal [25]. Taken together, ALN is a meritorious treatment for osteoporosis caused by estrogen deficiency, but whether ALN can simultaneously reverse the loss of bone strength caused by CBZ and estrogen deficiency, effectively inducing bone regeneration, still remains elusive [25, 26].

There have been countless studies on secondary osteoporosis and the impact of anti-osteoporosis drugs as a treatment over the past few years; however, little is known specifically about the use of anti-osteoporosis drugs in AED-related osteoporosis [15, 22, 27, 28]. We hypothesized that ALN might promote femoral metaphyseal defect bone regeneration in rats with estrogen deficiency when treated with CBZ. Subsequently, we investigated the effect of ALN on femoral bony deficits in ovariectomized rats under the action of CBZ.

## Methods

One hundred, 12-week-old female SD rats, weighing 210–230 g, were kept in animal cages with free access to food or water at 20–24 °C and 45–60% humidity. The experimental rats were exposed to 12 h light alternating with 12 h darkness. The present study was conducted in accordance with guidelines for the raising and use of experimental animals issued by the Chinese Society for Experimental Animals. This program was approved by the medical ethics review committee of Wannan Medical College. Operations were performed under pentobarbital sodium anesthesia administered by an experienced operator with a concerted effort to reduce pain in the animals. The researchers optimized their procedures to ensure animal welfare.

**Fig. 1:**
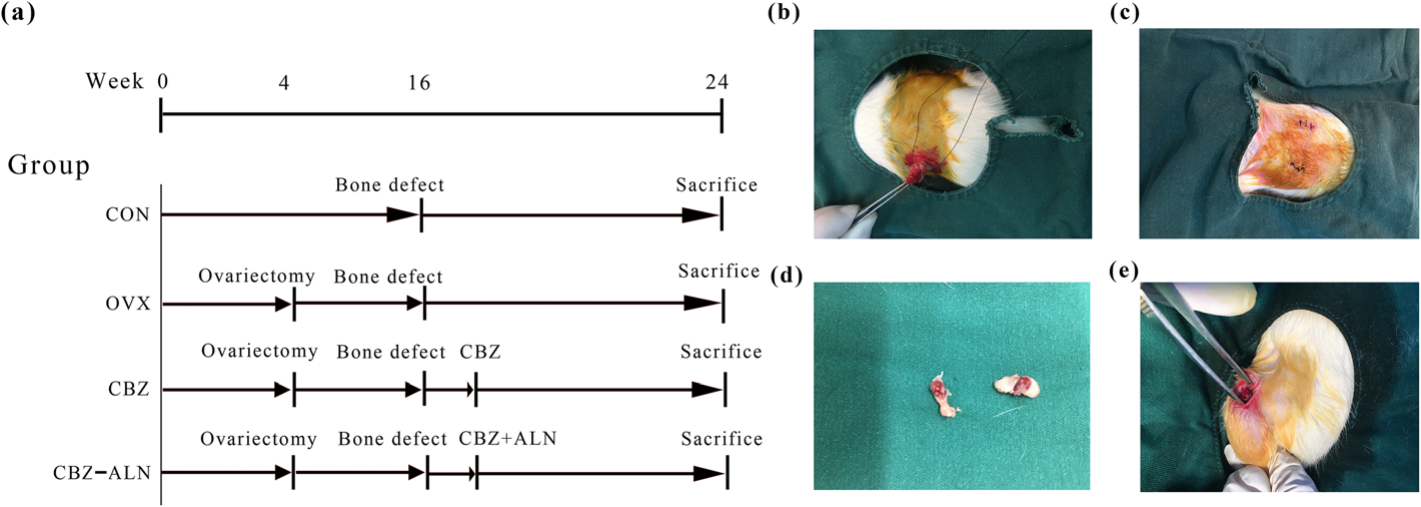
**a** The specific steps of this experiment. Ovariectomy (**b**–**d**) and femoral metaphyseal defect surgery (**e**) were conducted in female SD rats (**a**) Flow chart describing the study design; (**b-e**) ovariectomy and surgery of femoral metaphyseal defect conducted in female SD rats

The experimental timeline is summarized in Fig. 1. After sham operation (*n* = 30) or bilateral ovariectomy (*n* = 70) according to previous reports [29], it takes about a trimester before a surgery of bone defect to establish the osteoporosis model. Ten randomly selected ovariectomized rats and the ten sham-operated rats were sacrificed. Subsequently, femoral samples were collected for bone mineral density (BMD), high-resolution micro-CT, and histomorphometric analysis to verify the establishment of osteoporosis model.

The sample size of the present study was calculated according to earlier researches [30–33]. Effect size was calculated as 0.335. The power of this experiment was taken to be 80% at α = 0.05 and 95% confidence interval using a software of G*power v3.1.9.2. Finally, the sample size was calculated to be 18 rats/group. Considering the possible mortality of rats over a longer period of time, the present study included 20 animals in each group.

**Fig. 2:**
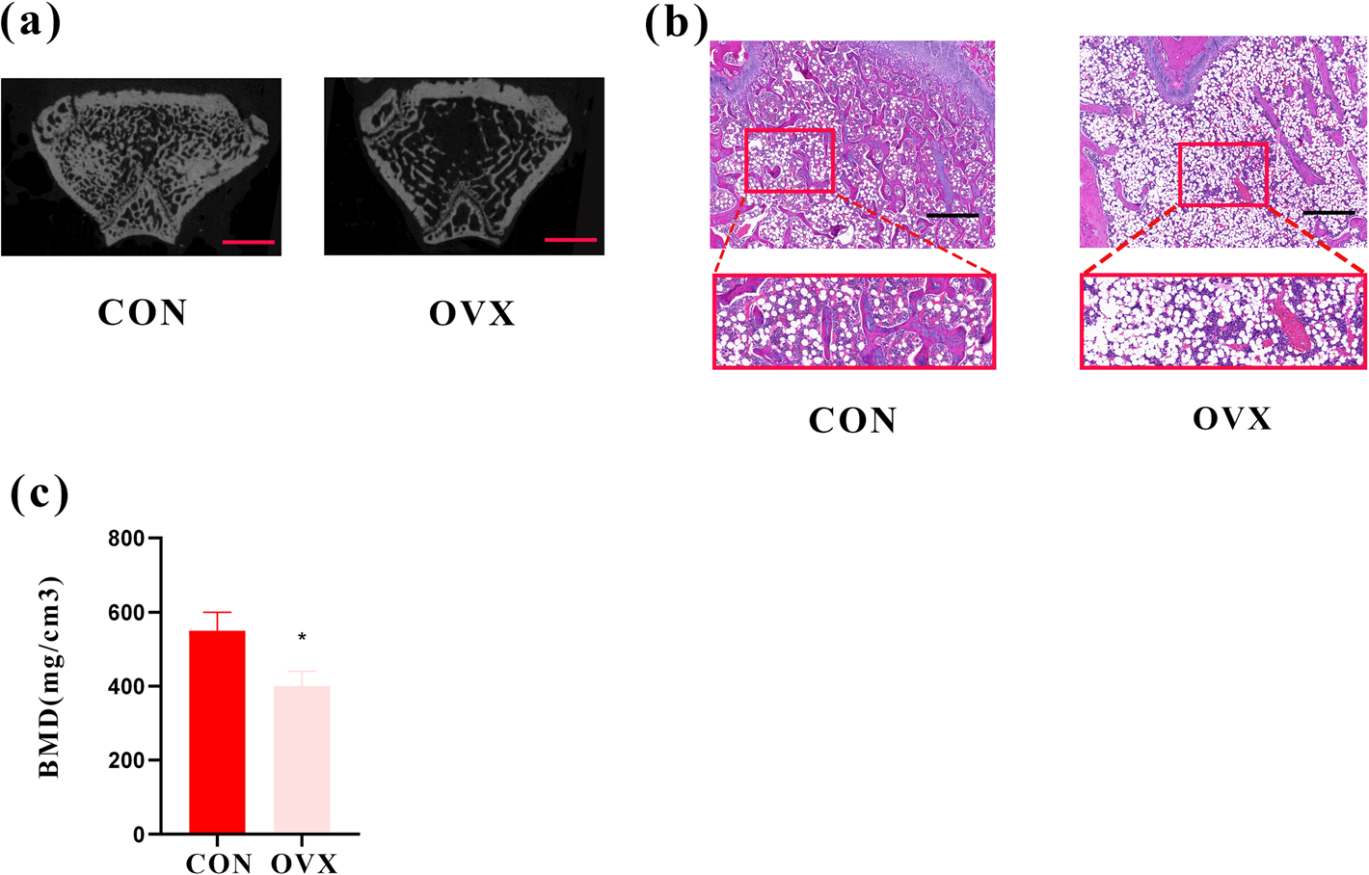
**a** Two-dimensional representation of normal bone in CON group and osteoporotic bone in OVX group (bar 2 mm). **b** Hematoxylin-eosin staining for CON and OVX groups. (magnification of 40, bar = 200 μm) **c** Comparison of bone density of trabecular bone between the group of CON and OVX. *P < 0.05 compared with the CON group

Then, the acclimatized rats underwent either bilateral laparotomy (CON, *n* = 20) or bilateral ovariectomy (*n* = 60). After 12 weeks, the ovariectomized rats were randomly divided into three groups: OVX rats with sham operation (OVX, *n* = 20), OVX rats with carbamazepine (CBZ, CBZ 75 mg/kg/day, *n* = 20), or OVX rats with carbamazepine and alendronate (CBZ-ALN, CBZ 75 mg/kg/day, ALN 2 mg/kg/day, *n* = 20) by intragastric gavage. The dosage for CBZ and ALN used in this study has been described previously [22, 31, 34]. A small DC drill was used under 0.9% sodium chloride to create a cylindrical defect with a diameter of 1.5 mm and a penetration length of about 4 mm. The femoral bony deficit is located slightly below the distal epiphyseal growth plate as described earlier [35] (Fig. 2). All rats were sacrificed after 8 weeks. Prior to the operation, all of the instruments were sterilized with high temperature, and rats were fasted for 12–14 h. The dosage of these drugs is adjusted according to the weight measured in rats per week. Eight weeks after the bone defect model was developed, all living rats in each group were euthanized. Serum samples were collected from the rat caudal artery and stored at − 80 °C until bone conversion biochemical indicators were assessed. The right distal femurs were taken and preserved before biomechanical testing, histological assessment, micro-computed tomography (micro-CT), and immunohistochemical evaluation (IHC).

### Micro-CT evaluation

Each femur was cut into two sections, and the distal portion was preserved. All specimens of femoral tissue were fixed and stored in 10% neutral formalin for at least 48 h under constant ambient temperature. A high-resolution micro-CT (μCT 100, Scanco Medical AG, Brüttisellen, Switzerland) was used to measure the primary microstructure of the defect area in the femur specimen. The X-ray tube was set at 70 kV, 200 μA with a 300 ms exposure time. Subsequently, the obtained greyscale images with a resolution of 15 μm were reconstructed and analyzed using the Scanco software. In order to assess bone regeneration in the area of the defects, the region of interest (VIO) was defined as the center of each bone defect, and a selected area with a diameter of 1.5 mm was evaluated layer by layer to avoid the inclusion of native bone edges. The measurements of primary bone structural indices were assessed from the high-resolution μ-CT images for all specimens: bone volume/tissue volume (BV/TV, 1), bone mineral density (BMD, mg HA/cm^3^), trabecular thickness (Tb.Th, mm), trabecular number (Tb.N, 1/mm), and trabecular spacing (Tb.Sp, mm).

### The hematoxylin/eosin (H&E) staining and Masson’s trichrome staining

Samples were immersed in fresh 10% neutral formalin for 72 h at ambient temperature. Femur specimens were decalcified in 15% neutral EDTA buffer for 3 months. After the decalcification was completed, the decalcified specimens were sequentially placed in different concentrations of ethanol for a gradient dehydration. The bones were then embedded in methyl methacrylate and cut into 5-mm sections for hematoxylin and eosin (H&E) and Masson’s staining [36]. All the samples were prepared and examined in a blind manner to reduce experimenter bias. New bone formation area (%) = new bone formation area/bone defect area × 100%.

### Biomechanical testing

Compression testing was performed on the metaphysis of the distal femurs according to the protocol previously described [37]. Briefly, the distal end of the dorsal femur was placed in an aluminum alloy base attached to the mechanical testing system. This position keeps the femur in a steady three-point contact with the base, preventing sliding during the process of fracture testing. Then, a 1 N preload was applied to the condyle side of the femur, and compression was initiated at 2 mm/min until bone failure. The ultimate load at failure was considered as the strength of the femoral condyles.

### Bone turnover markers

All the sample of serum were separated from the starved rats for subsequent analysis. For markers of bone formation, ELISA kit (Immunodiagnostic Systems, UK) was used to measure the levels of N-terminal propeptide of type I procollagen (PINP) and osteocalcin (OCN). The levels of bone resorption marker including C-terminal crosslinked telopeptide of type I collagen (CTX-1) was then examined via ELISA kit (Biomedical Technologies, USA) according to the manufacturers’ specifications.

### Immunohistochemical evaluation

Immunohistochemical staining was performed according to the methods previously described [36]. The demineralized and paraffin-embedded femurs were cut into 5-μm sections. Sections were then treated with dimethylbenzene, followed by a gradient descent of alcohol from 100 to 70%, followed by incubation in an antigen retrieval solution and finally hydrogen peroxide. All of the sections were incubated in the prediluted monoclonal primary antibody [OCN (1:200, Abcam), Col I (1:100, Abcam), Tracp-5b (1:120, Abcam)] overnight at 4 °C. Non-immune goat serum was used to replace the primary antibody in the negative controls. All slides were incubated overnight with secondary antibodies, washed, and stained with 3,3′ diaminobenzidine tetrahydrochloride and hematoxylin. The results of IHC staining were quantified with the IPP 6.0 software, and the positive staining was expressed as percentage of the positive area.

### Statistical analyses

All values were presented as the mean ± standard deviation (SD). Statistical analyses were performed using the statistics package SPSS 22.0 (IBM, Armonk, New York). Comparisons between different groups were analyzed by using one-way ANOVA and Tukey’s post hoc test. A *P* value less than 0.05 was considered statistically significant for all analyses.

## Results

### Authentication of successful establishment of the OVX rat model

The histological and high-resolution micro-CT images of the femoral head are shown in the Fig. 2a and b. Before the surgery of bone defect, the bone mineral density of the distal femur from rats in the CON group was 37.6% higher than the OVX group (*P* < 0.05) (Fig. 2c). All results above suggest the establishment of osteoporosis in female ovariectomized Sprague Dawley rats.

### Microstructural parameters

**Fig. 3:**
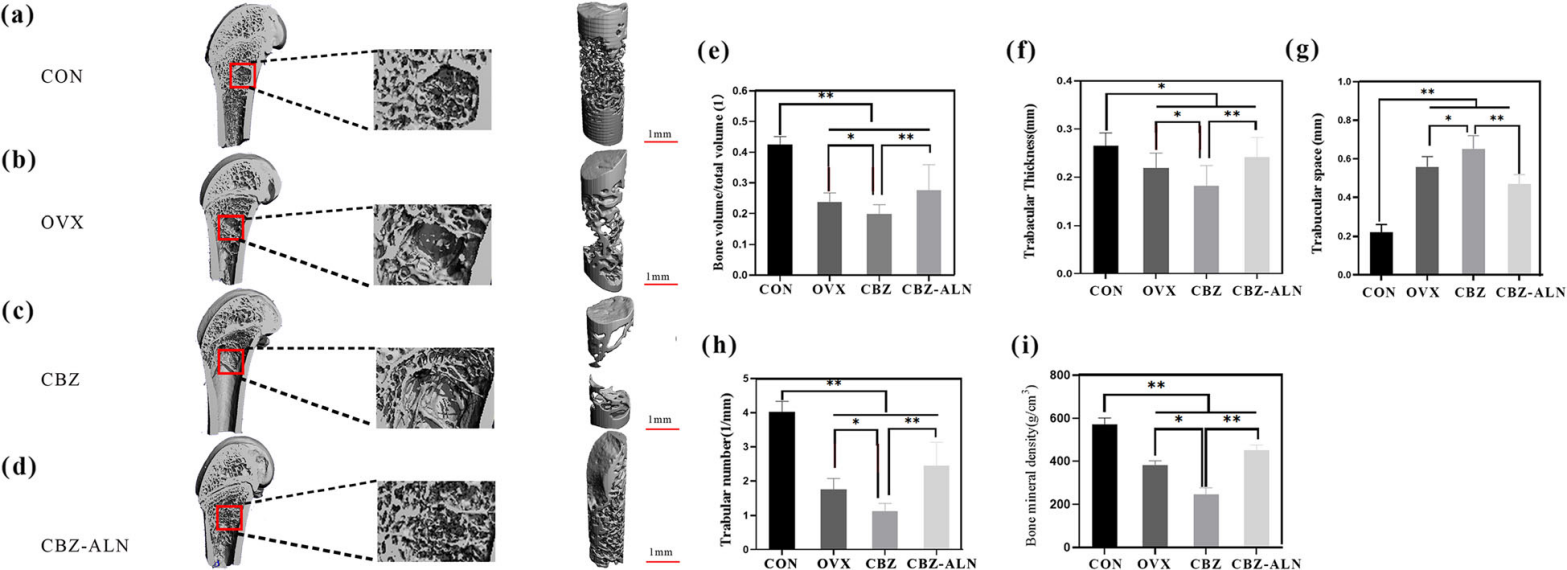
Three-dimensional reconstruction of femoral defect area in four groups in experiment rats after 8 weeks (**a**–**d**). Quantitative results of high resolution micro-CT evaluation 8 weeks after bone defect operation (**e**–**i**). *N* = 20 specimens per group. Data were displayed as mean ± standard deviation. Error bars in the figure indicated as standard deviation. *P < 0.05, **P < 0.01

The three-dimensional reconstruction images of trabeculae and quantitative results of each group are shown in Fig. 3. After 8 weeks, compared with the CON group, the microparameters of Tb.N, Tb.Th, BV/TV, and BMD in OVX rats significantly decreased. Besides, compared to the group of CON, bone neoformation around bone defect in OVX rats was obviously less, with a significant decrease of BV/TV, Tb.Th, and Tb.N as well as increased Tb.Sp of the new bone. In OVX rats, CBZ treatment further deteriorated bone microparameters. However, CBZ-ALN group significantly improved the bone neoformation around the bone defects indicated by the higher parameters and features of the newly formed bone as compared to the group of CBZ (*P* < 0.05).

### Histological analyses

**Fig. 4:**
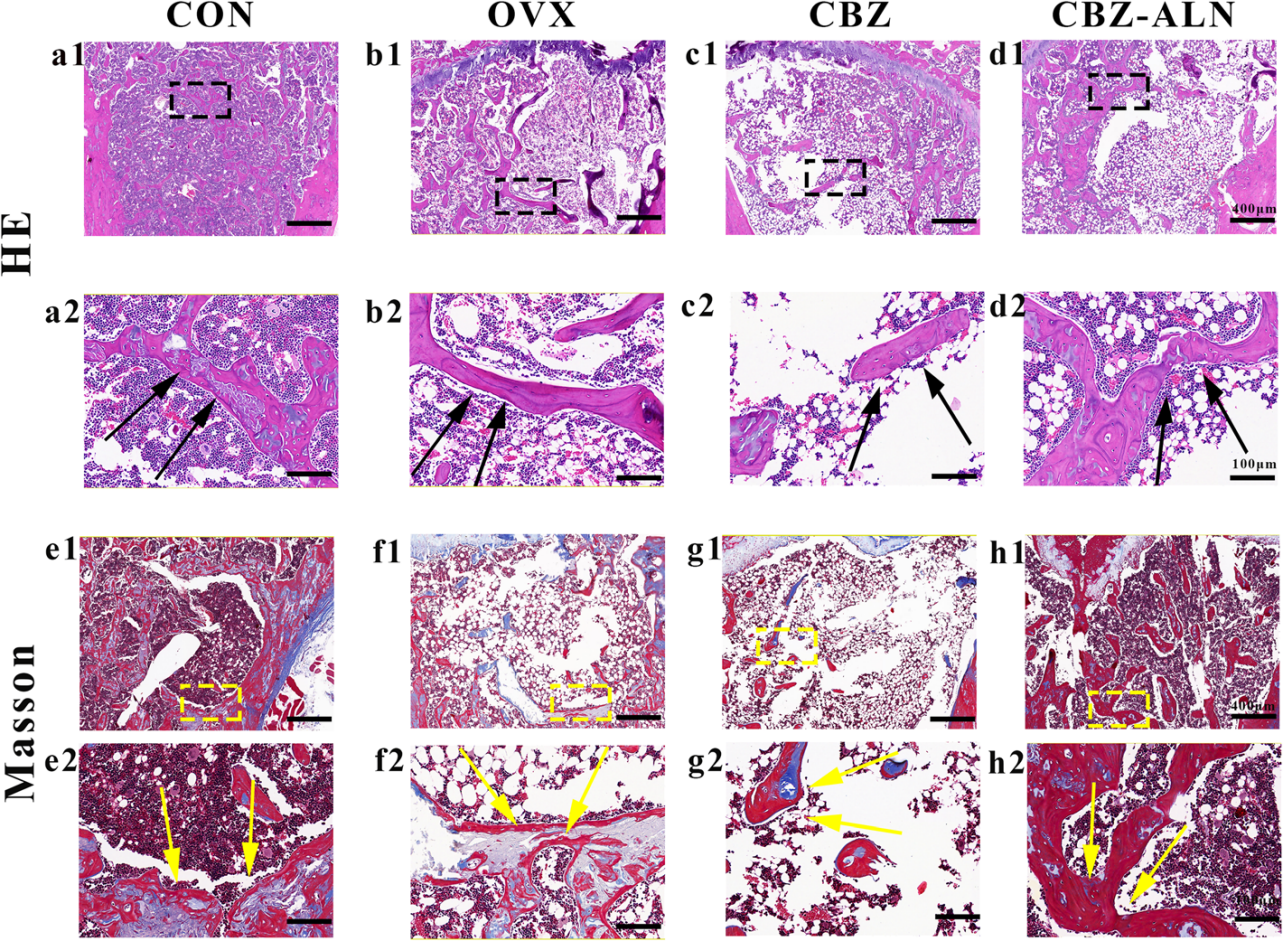
H&E staining used for the analysis of the histomorphology of the new bone around different groups: CON (**a1**), OVX (**b1**), and CBZ (**c1**), CBZ-ALN (**d1**). The black rectangle represents the location of new bone formation around the bony deficits. (black arrow: new bone, bar 400 mm). Masson’s trichrome staining used for the analysis of new bone formation: CON group (**e1**), OVX group (**f1**), and CBZ group (**g1**), CBZ-ALN group (**d3**). The yellow rectangle represents the location of new bone formation around the bony deficits. Enlarged view specifically shows new bone formation in the corresponding square: CON (**e1**), OVX (**f1**), CBZ (**g1**), and CBZ-ALN (**h1**) for H&E staining and CON (**e2**), OVX (**f2**), CBZ (**g2**), and CBZ-ALN (**h2**) for Masson’s dyeing (yellow arrow: new bone, bar 100 mm)

Consistent with the results of the high-resolution micro-CT, H&E and Masson’s trichrome staining showed increased bone trabecular loss in the OVX groups compared to CON group at 21 weeks after bilateral ovariectomy. Compared with the group of CON, there was less bone neoformation in the defect area of ovariectomized rats. In addition, the new bone formation in CBZ-ALN group was larger than that in the OVX and CBZ groups when compared with the CON group and CBZ group. For the CBZ-ALN group, more bone neoformation occurred in the largest defect site from the defect edge to the center compared with OVX and CBZ groups (Fig. 4).

### Biomechanical testing

**Fig. 5:**
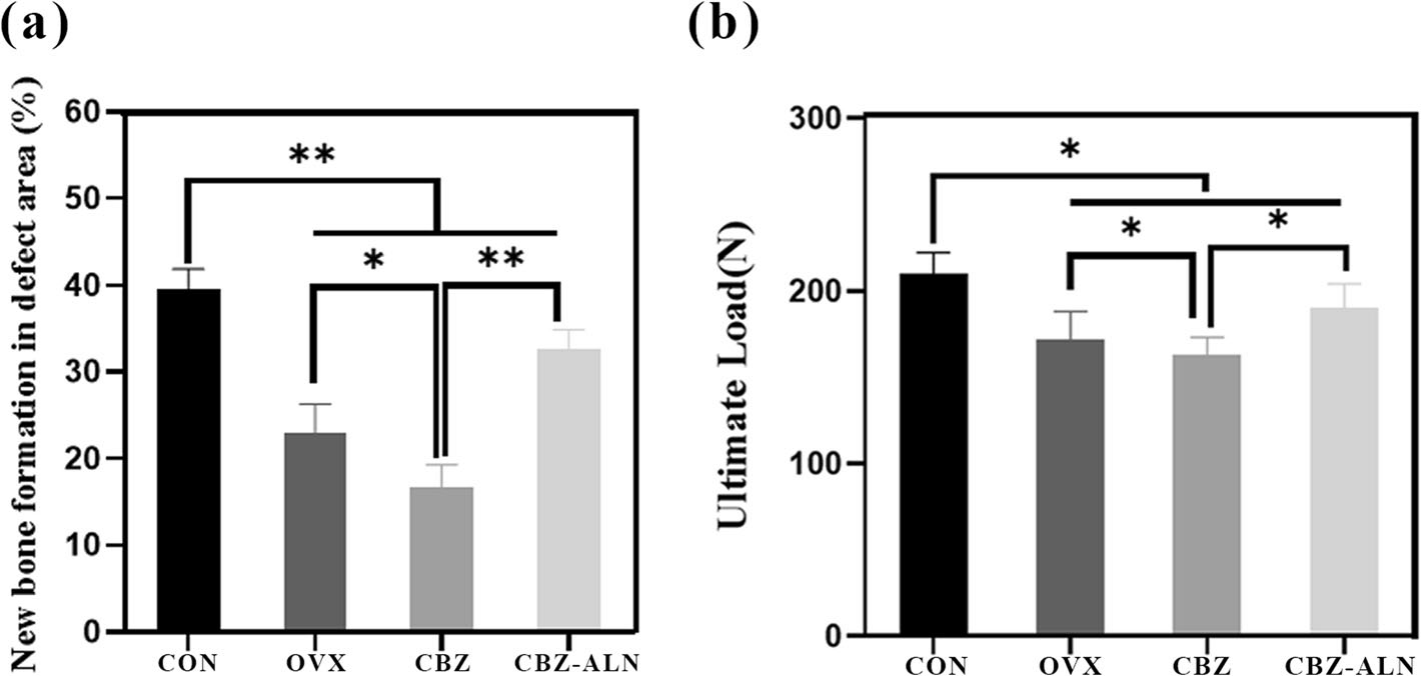
**a** Histometric analysis for new bone area of the H&E staining. Data were expressed as mean ± SD; error bars in the figure indicate SD. *P < 0.05, **P < 0.01. **b** Results of biomechanical were expressed as maximal push-out force (N) for all groups. *N* = 20 specimens/group. Data were expressed as mean ± SD; error bars in the figure indicate SD. *P < 0.05

The biomechanical test results of the femoral condyle are expressed as ultimate load (Fig. 5). Eight weeks after surgery of the bone defect, the ultimate load in ovariectomized rats was significantly lower than in the CON group. The limit load of the CBZ-ALN group was notably higher than the OVX group and the CBZ group, while the CBZ group represented the minimum ultimate. There were significant differences observed among the four groups (*P* < 0.05).

### Bone turnover markers

**Fig. 6:**
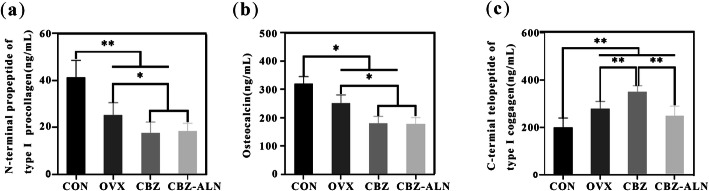
Quantification of bone turnover markers (*N* = 30). Procollagen type I N-terminal propeptide (PINP) (**a**), osteocalcin (OCN) (**b**) and C-terminal telopeptide collagen (CTX-I). Data were expressed as mean ± SD; error bars in the figure indicate SD. *P < 0.05, **P < 0.01

The results of serological analysis are shown in Fig. 6. Decreased levels of OCN and P1NP in OVX, CBZ, and CBZ-ALN groups were statistically significant compared to the CON group. There was no significant difference in OCN and P1NP indicators between CBZ and CBZ-ALN groups (*P* < 0.05), but significantly lower than that of the group of OVX. A significant increase in CTX-1 levels was observed in ovariectomized animals as compared to the group of CON. However, CBZ group presented statistically significant higher values than OVX group and CBZ-ALN group. Besides, CTX-1 serum levels in the CBZ-ALN group were significantly lower than those in the other two groups (*P* < 0.05).

### Immunohistochemical analysis

**Fig. 7:**
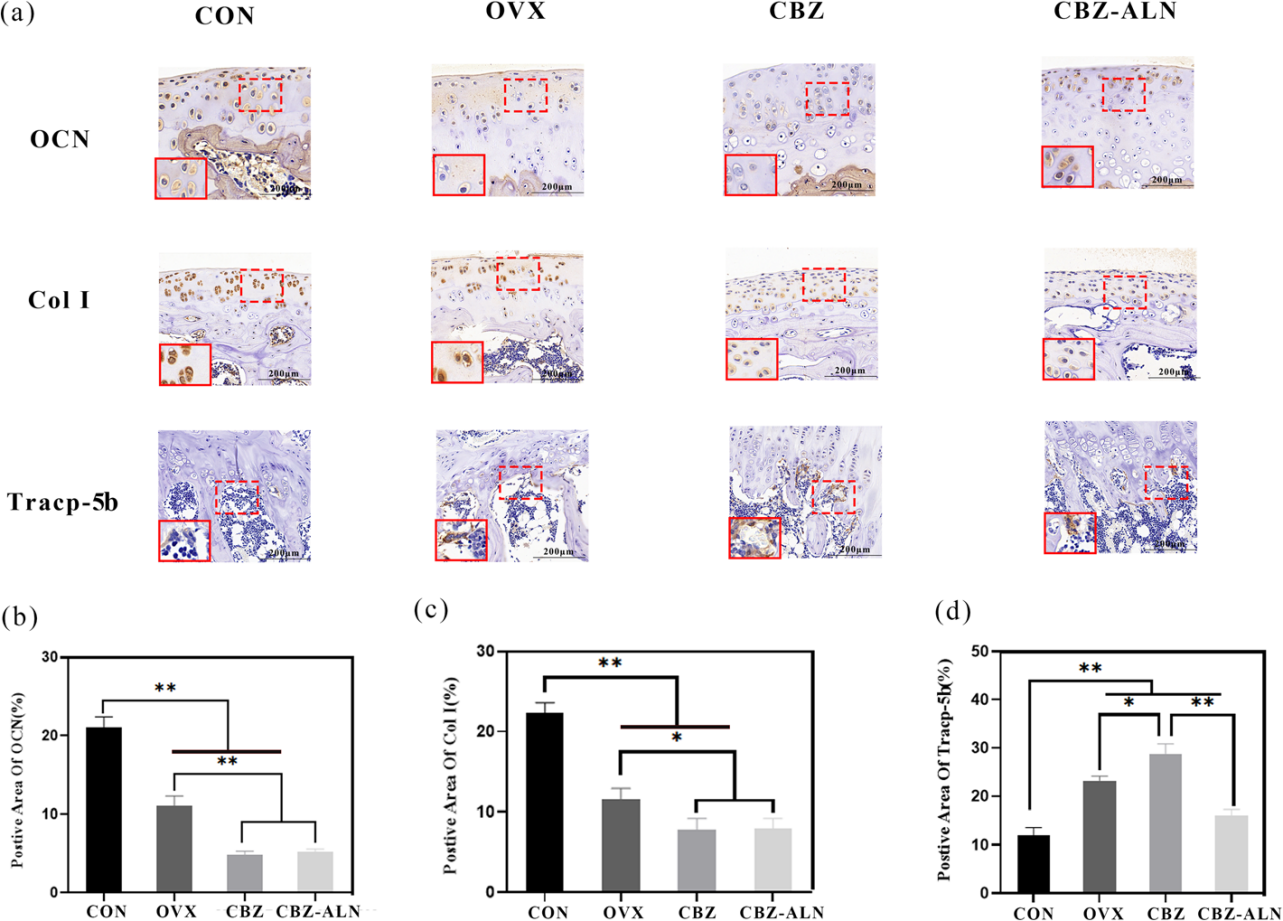
Immunohistochemical staining of osteocalcin (OCN), collagen I (Col I), and tartrate-resistant acid phosphatase (Tracp-5b) were used to observe osteogenesis in femur distal defects **a** Deptor expression of CON and OVX rats evaluated by IHC (*N* = 20). The red dotted rectangles indicate positive areas while solid rectangles refer to a higher magnification of immunoreactivity positive areas. Magnification was × 200. Scale bars 200 μm. **b** Immunohistochemistry quantitative analyses for OCN, Col I, and Tracp-5b in the bone defects among the 4 groups. Data are expressed as mean ± SD. *P < 0.05, **P < 0.01

The protein expression of the experimental groups is shown in Fig. 7. The Col I and OCN values of ovariectomized rats in three groups were statistically different from those in CON group (*P* < 0.05). In addition, OCN and Col I expression in CBZ group was significantly lower than that in CON and OVX groups (*P* < 0.05). However, no significant differences in OCN and Col I staining were found between CBZ and CBZ-ALN groups at 8 weeks after bone defect surgery (*P* > 0.05). For the expression of Tracp-5b, a significant increase was discernible in all ovariectomized animals 20 weeks post-OVX. However, Tracp-5b expression further increased when treated with CBZ. Statistical analysis showed that the Tracp-5b value of CBZ-ALN group was significantly lower than that of OVX and CBZ groups (*P* < 0.05).

## Discussion

In this experiment, bone defect surgery for the standard osteoporotic animal model after bilateral ovarian surgery was conducted at 12 weeks. The bone neoformation of the distal metaphyseal of the femur was then assessed in all surviving rats over an 8-week period. In this trial, histological and CT analysis confirmed that preexisting estrogen deficiency leads to delayed bone neoformation of femoral metaphyseal defect while single-dose of CBZ further delays the process of bone regeneration. In addition, the results of this experiment indicated that ALN has a positive effect on local bone neoformation in rats with osteoporosis which can reverse the negative impact of CBZ on bone mass.

The most important pathogenesis of postmenopausal osteoporosis is the significant increase in random remodeling leading to bone loss, serious deterioration of bone microstructure, and bone quality decline [38]. The increase in bone matrix remodeling is related to the lack of estrogen during menopause. Ovariectomized rats are a well-established animal model that mimics the conditions of postmenopausal females and has been used in a variety of studies in osteoporosis [5, 39]. This animal model requires 3 months in order to induce osteoporosis in rats. In line with these expectations, a significant decrease in the bone density of the distal femur was found 3 months after initiation.

In post-menopausal females, estrogen deficiency induces the expression of the interleukin-6 (IL-6) in the early phases after bone defect appear reducing the differentiation ability of the osteogenic system which leads to delayed bone neoformation [40]. Further study suggest that reduced estrogen levels resulting from ovariectomies lead to an increase in osteoclast activity, which results in a decrease in the volume of the trabecular bone [41]. This may be due to estrogen deficiency that enhances bone resorption by increasing receptor activator of NF-КB ligand (RANKL) and by adding sclerostin to reduce bone formation [22]. In addition, it has been confirmed that when epilepsy and osteoporosis occur simultaneously, the bone mass of patients which is already severely low, will worsen by the use of antiepileptic drugs [10]. In in vitro experiments, we observed that microstructural parameters such as BV/TV and Tb.Th in OVX rats treated with CBZ were significantly lower than the OVX and CBZ-ALN treatment groups. These data indicated that the coexistence of estrogen deficiency and CBZ treatment delayed bone neoformation in the rats, indicated by the lower BMD, BV, and BV/TV, as well as an increase in Tracp-5b expression. The results suggest that the preexistence of estrogen deficiency can seriously affect the repair of femoral metaphyseal defect by reducing bone formation and increasing bone resorptive activity. It is widely accepted that abnormal formation and activation of osteoclasts play a pivotal role in osteolysis under pathological conditions [42–44]. AEDS such as CBZ can promote the catabolism of 3,17-dihydroxyestratriene and the composition of sex hormone-binding globulin (SHBG). The increase levels of SHBG will lead to a decrease in serum C19 steroid levels, resulting in a decrease of C19 androgen conversion and the formation of estrogen [45]. A reduction in estrogen promotes the production of IL-7 in the bone tissue. As a result, T cells are further activated to release interferon (IFN), tumor necrosis factor (TNF), and RANKL, leading to the formation of osteoclasts [8]. Alendronate (ALN) is a nitrogen-containing bisphosphonate concentrated at the site of active bone remodeling in vivo. It can inhibit bone resorption by influencing the formation of the boundary of osteoclast wrinkles and regulating the molecular pathway of cytoskeleton. When clinicians treat osteoporosis, alendronate is often considered because it has been shown to prevent bone loss by rapidly inhibiting osteoclasts [23, 24]. When postmenopausal osteoporosis and epilepsy occur concurrently, bone conversion becomes abnormally high, and it is exacerbated by the use of anti-epileptics [10, 16, 46]. However, the question of whether to add bisphosphate to antiepileptic drugs remains to be addressed by clinicians [20].

Moreover, we assessed the P1NP, OCN, and CTX-1 serum levels in all surviving experimental rats. P1NP and OCN had their levels significantly decreased in OVX rats compared with the group of CON. Besides, P1NP and OCN levels further decreased upon treatment with CBZ. However, no significant difference were discernible between CBZ and CBZ-ALN groups. All above results indicate that osteoporosis significantly reduces the level of bone formation markers, and CBZ treatment further reduces these markers. At the same time, a significant increase in CTX-1 levels was observed in ovariectomized animals as compared to the group of CON. However, CBZ group presented statistically significant higher values than OVX group and CBZ-ALN group while serum CTX-1 levels decreased significantly following CBZ-ALN treatment. These results suggest that ALN affects bone resorption markers and thus maintains osteoclast activity inhibition.

In order to understand the mechanisms related to bone formation and bone resorption, immunostaining for OCN, Col I, and Tracp-5b was performed. In this in vitro study, it was found that the expression of OCN and Col I was significantly decreased in the distal femoral of ovariectomized rats compared with the group of CON. No significant differences were discernible between OCN and Col-1 expression levels of CBZ and CBZ-ALN groups. In addition, in the investigation of the inhibitory effect on osteoclast activity, the expression of Tracp-5b was analyzed. Strikingly, we found that ALN treatment reduced osteoclast activity remarkably. Alendronate (ALN) is a nitrogenous bisphosphonate which can prevent bone resorption. ALN therapy increases bone strength by significantly affecting bone mass and bone quality [23, 47]. Recently, experimental data obtained by Oliveira et al. [31] documented that using alendronate to treat craniofacial defects showed stronger bone regeneration in ovariectomized rats. Similar results were obtained by another research group [47]. More recent results indicate that in order to prevent BMD loss after AED, vitamin D and/or possible bisphosphonates are recommended. Bone mass in growing rats did not decrease when another antiepileptic drug, valproic acid sodium (VPA), is combined with the active form of bisphosphonates (ALN). However, due to the limited clinical data, whether or not to add bisphosphonates still bothers clinicians [16]. In our study, we demonstrated that under the negative effect of CBZ, ALN presented a strong bone neoformation, enhanced bone mass, and reduced osteoclast activity compared with single-dose of CBZ in this model.

To the best of our knowledge, this is the first report describing the effects of CBZ and ALN on femoral metaphyseal defect of osteoporotic rats. This is a significant finding leading to promising further treatment protocols for enhanced bone neoformation. In the first place, a moderate number of rats mean that the power of this experiment to attest statistical differences was relatively low. Another limitation of the study is that although rat bone is often used in bone repair models, rat bones lack the histology of the Haversian bone system. Compared with humans, how this affects the pathophysiological pathways of bone injury and healing remains unclear. Whether the lack of a Haversian bone system alters the local response and nutrient or waste shuttling in the pathological process is also unknown. In addition, other limitations in rat compared to human bone includes decreased size and elasticity [48]. Therefore, this work cannot be directly used in clinical practice at this time. Besides, the condition was not observed after 8 weeks since the experiment ended at the 8-week mark. Moreover, the effects of CBZ-ALN in in vitro cell research was not conducted, nor was an older animal model considered, which might better represent elderly osteoporotic bone. Hence, further investigation is necessary.

## Conclusion

The findings presented here suggest that CBZ treatment deteriorated the microarchitectural properties of bone and decreased bone mineral density in ovariectomized rats. Notably, in our study, ALN caused a strong inhibition of bone turnover and an increase in bone density and bone neoformation though treated with CBZ. Although the specific mechanism by which ALN reverses CBZ was not fully elucidated, our results provide partial evidence that ALN would be promising as a method to achieve bone neoformation in femoral bony deficits to reverse the negative impacts of CBZ.

## Data Availability

All data generated or analyzed during this study are included in this published article.

## Abbreviations

OP: Osteoporosis
ALN: Alendronate
CBZ: Carbamazepine
AEDS: Antiepileptic drugs
OVX: Ovariectomy
OCN: Osteocalcin
RANKL: Receptor activator of NF-КB ligand
Col I: Type I collagen
Tracp 5b: Tartrate-resistant acid phosphatase
